# The equilibrium and kinetic drug binding properties of the mouse P-gp1a and P-gp1b P-glycoproteins are similar

**DOI:** 10.1038/sj.bjc.6690764

**Published:** 1999-11

**Authors:** J C Taylor, D R Ferry, C F Higgins, R Callaghan

**Affiliations:** Nuffield Department of Clinical Biochemistry & Cellular Science, University of Oxford, John Radcliffe Hospital, Oxford OX3 9DU, UK; Department of Clinical Oncology, Queen Elizabeth Hospital, Edgbaston, Birmingham, UK; MRC Clinical Sciences Centre, Imperial College School of Medicine, Hammersmith Hospital, London, UK

**Keywords:** P-glycoprotein, MDR, drug binding, steroid hormones

## Abstract

The gene encoding the multidrug resistance P-glycoprotein (P-gp) is duplicated in rodent species and the functional basis for this remains unresolved. Despite a high sequence similarity, the mouse P-gp1a and P-gp1b isoforms show distinct patterns of tissue distribution which suggest a specific role of the P-gp1b isoform in steroid transport. In the present study possible biochemical differences between the isoforms were directly investigated at the level of drug interaction. There was no detectable difference in the affinity or binding capacity of the two isoforms towards [^3^H]vinblastine at equilibrium. Similarly, the rate at which [^3^H]vinblastine associates with P-gp was indistinguishable between the two isoforms. Some modest differences were observed in the relative abilities of the multidrug-resistant (MDR) reversing agents CP100-356, nicardipine and verapamil to displace equilibrium [^3^H]vinblastine binding to P-gp1a and P-gp1b. The steroid hormone progesterone displayed a low affinity (*K*_i_ = 1.2 ± 0.2 μM for P-gp1a and 3.5 ± 0.5 μM for P-gp1b), suggesting an unlikely role as a physiological substrate. Thus the mouse isoforms do not appear to exhibit functional differences at the level of initial substrate interaction with protein. © 1999 Cancer Research Campaign


*British Journal of Cancer* (1999) **81**(5), 783–789
© 1999 Cancer Research Campaign
Article no. bjoc.1999.0764

### The equilibrium and kinetic drug binding properties of the mouse P-gp1a and P-gp1b P-glycoproteins are similar

**JC Taylor[1], DR Ferry[2], CF Higgins[3] and R Callaghan[1]**

[1]Nuffield Department of Clinical Biochemistry & Cellular Science, University of Oxford, John Radcliffe Hospital, Oxford OX3 9DU, UK; [2]Department of Clinical Oncology, Queen Elizabeth Hospital, Edgbaston, Birmingham, UK; [3]MRC Clinical Sciences Centre, Imperial College School of Medicine, Hammersmith Hospital, London, UK.

**Summary** The gene encoding the multidrug resistance P-glycoprotein (P-gp) is duplicated in rodent species and the functional basis for this remains unresolved. Despite a high sequence similarity, the mouse P-gp1a and P-gp1b isoforms show distinct patterns of tissue distribution which suggest a specific role of the P-gp1b isoform in steroid transport. In the present study possible biochemical differences between the isoforms were directly investigated at the level of drug interaction. There was no detectable difference in the affinity or binding capacity of the two isoforms towards [$^3$H]vinblastine at equilibrium. Similarly, the rate at which [$^3$H]vinblastine associates with P-gp was indistinguishable between the two isoforms. Some modest differences were observed in the relative abilities of the multidrug-resistant (MDR) reversing agents CP100-356, nicardipine and verapamil to displace equilibrium [$^3$H]vinblastine binding to P-gp1a and P-gp1b. The steroid hormone progesterone displayed a low affinity ($K_i = 1.2 \pm 0.2$ μM for P-gp1a and $3.5 \pm 0.5$ μM for P-gp1b), suggesting an unlikely role as a physiological substrate. Thus the mouse isoforms do not appear to exhibit functional differences at the level of initial substrate interaction with protein.
© 1999 Cancer Research Campaign

**Keywords:** P-glycoprotein; MDR; drug binding; steroid hormones

Tumour cells expressing P-glycoprotein (P-gp) display resistance to a wide range of chemically and structurally unrelated chemotherapeutic drugs (reviewed by (Gottesman and Pastan, 1988). P-gp is localized to the plasma membrane and utilizes the energy of ATP hydrolysis to efflux drugs from cells, thereby maintaining their intracellular concentrations below cytotoxic levels. In humans, the drug-resistance P-gp is encoded by a single gene (*MDR1*), but in the mouse and other rodents the equivalent gene is duplicated (*mdr1a* and *mdr1b*) (Gros et al, 1986; Devault and Gros, 1990). The mouse P-gps are both 1276 amino acids in length but have different apparent molecular weights (160 kDa and 180 kDa for P-gp1a and P-gp1b respectively), due to non-equivalent glycosylation. The two murine proteins share 84% amino acid identity, although the linker region which contains several sites for phosphorylation by protein kinase C (PKC) is the least conserved region between the two isoforms (Devault and Gros, 1990).

Despite their high level of sequence identity, the complementary patterns of tissue-specific expression suggest that the two rodent isoforms may perform distinct functions (Croop et al, 1989). P-gp1a is expressed at high levels in the intestine, liver and testis, and also in the lung and brain where it is thought to contribute to the blood–brain barrier (Schinkel et al, 1994). In contrast, P-gp1b is expressed in tissues associated with steroid biosynthesis and distribution, such as the adrenal gland and ovary, and is induced specifically in the uterus during pregnancy (Arceci et al, 1988; Trezise et al, 1992). The steroid hormone progesterone increases during pregnancy, and the *mdr1b* gene, but not the *mdr1a* gene, is regulated by a progesterone response element in its promoter (Piekarz et al, 1993). Direct evidence that the P-gp1b isoform can transport steroids comes from the observation that disruption of the *mdr1b* gene in mouse adrenal cells is associated with decreased adrenocorticotropic hormone-stimulated steroid secretion (Altuvia et al, 1993). Taken together, these observations have given rise to the suggestion that the P-gp1b isoform is involved in the translocation of steroids such as progesterone.

A direct interaction of several steroid hormones with P-gp from a variety of tissues and sources has been demonstrated in vivo. More specifically, progesterone inhibits vinblastine binding and azidopine photolabelling of both of the mouse P-gp isoforms in vitro (Yang et al, 1990), showing that both isoforms are capable of interacting with steroids. However, if the distinct tissue-specific patterns of expression of P-gp1a and P-gp1b reflect differences in function related to steroid transport, it is important to show directly that the two proteins are functionally distinct. Some indications of differences in the biochemical properties of the P-gp1a and P-gp1b isoforms have been reported: (i) lower progesterone concentrations are required to inhibit transport by P-gp1b than by P-gp1a (Yang et al, 1990); (ii) P-gp1a and P-gp1b differ in their ability to confer cellular resistance to several drugs, including colchicine and actinomycin D (Devault and Gros, 1990; Yang et al, 1990; Kajiji et al, 1993; Tang-Wai et al, 1995); (iii) a variety of P-gp modulators were reported to exhibit isoform-specific differences in their capacity to reverse vinblastine resistance (Tang-Wai et al, 1995). However, these are indirect tests of substrate specificity and do not provide direct information on steroid-P-gp

*Received 5 January 1999*
*Revised 27 May 1999*
*Accepted 3 June 1999*

*Correspondence to:* R Callaghan interactions. Thus, if a role for P-gp1b in steroid transport is to be confirmed it is important to demonstrate that the two murine P-gp isoforms have different substrate interaction profiles.

In this study we have assayed directly the interaction of drugs with the two mouse isoforms. Equilibrium binding assays were used to assess substrate affinity, association kinetics and the potency of a range of modulators in displacing bound substrate. The drug binding characteristics of the two mouse isoforms were shown to be very similar. In particular, no differences in response to the modulator progesterone were apparent. These findings suggest that the different patterns of expression of the two P-gp isoforms are more likely to reflect an alternative function than differences in their ability to respond to progesterone. As P-gp1a and P-gp1b have previously been shown to differ in their abilities to modulate activation of cell-swelling activated chloride currents (Valverde et al, 1996), the possibility that this difference may account for the duplication of isoforms is discussed.

### METHODS

### Chemicals

[$^3$H]vinblastine sulphate (13.5 Ci mmol$^{-1}$) and [$^3$H]progesterone (95 Ci mmol$^{-1}$) were purchased from Amersham plc (UK). Vinblastine sulphate, progesterone, nicardipine and verapamil were obtained from Sigma Chemicals. CP100-356 was a generous gift from Pfizer. The monoclonal antibody against P-gp (C219) was purchased from Centocor Diagnostics. Ham's F12 medium was from Gibco. All other tissue culture reagents were provided by the ICRF Clare Hall Laboratory.

### Cell culture

Chinese hamster LR73 ovary cells, and their transfected drug-resistant derivatives overexpressing either *mdr1a* (LR73-1a) or *mdr1b* (LR73-1b) cDNAs were a kind gift from Dr P Gros (Montreal). Cells were grown as described previously (Devault and Gros, 1990). Drug-resistant lines were maintained in 50 ng ml$^{-1}$ vinblastine sulphate which is sufficient to prevent re-emergence of drug-sensitive cells ($EC_{50}$ = 10 ng ml$^{-1}$) but significantly less than the maximal resistance level of the cells ($EC_{50}$ = 200–250 ng ml$^{-1}$).

### Plasma membrane isolation

Plasma membrane fractions were isolated according to previously published methods (Lever, 1977). Briefly, $1 \times 10^9$ cells were grown to confluence, harvested, washed and re-suspended in ice-cold 50 mM Tris–HCl buffer pH 7.4, containing the protease inhibitors phenylmethylsulphonyl fluoride (PMSF; 1 mM), EDTA (1 mM) and benzamidine (1 mM). The cells were disrupted by nitrogen cavitation (1500 p.s.i.; $2 \times 15$ min) and the membrane fraction isolated by sucrose (35% w/v) density centrifugation. Membranes were resuspended and stored in buffer containing 0.01 M Tris–HCl, pH 7.4 and 0.25 M sucrose at –80°C.

### P-glycoprotein detection

The amount of P-gp in LR73, LR73-1a and LR73-1b membranes was assessed by Western immunoblot analysis, based on published methods (Towbin et al, 1979). Membranes were solubilized in 1% sodium dodecyl sulphate (SDS) and the protein concentration assayed with a modified Lowry assay (detergent-compatible Biorad kit) using bovine serum albumin as a standard. Proteins were electrophoretically separated on 7.5% SDS-polyacrylamide gels and transferred onto nitrocellulose membranes (Hybond; ECL). Membranes were incubated sequentially with the anti-P-gp monoclonal antibody C219, and horseradish-peroxidase (HRP)-linked secondary antibody and proteins detected by chemiluminescence. Lanes of serially-diluted total membrane protein (1–50 μg) were quantitated for P-gp by densitometry.

### Equilibrium binding of [$^3$H]vinblastine

Equilibrium drug binding to plasma membranes was assayed using a rapid filtration method, as described previously (Ferry et al, 1992). Membranes (50 μg) were incubated with a range of [$^3$H]vinblastine concentrations (0.1–100 nM) in buffer A (50 mM Tris–HCl, pH 7.4) in a total assay volume of 0.2 ml for 120 min in the dark. Three millilitres of ice-cold buffer B (20 mM Tris–HCl, 20 mM magnesium chloride pH 7.4) were then added to the samples, and bound and free drug separated by suction through Whatman GF/F and 0.2 μm nitrocellulose filters (pre-wetted with 0.1% bovine serum albumin (BSA) in buffer A) using a Millipore multichannel filtration manifold. Filters were washed twice with 3 ml ice-cold buffer B, and retained radioactivity quantitated by liquid scintillation counting. All results were corrected for non-specific binding which was defined as the amount of [$^3$H]vinblastine bound in the presence of an excess (3 μM) of unlabelled vinblastine.

Drug displacement assays employed 60 μg or 100 μg of total membrane protein for LR73-1a or LR73-1b membranes, respectively, and 15–20 nM [$^3$H]vinblastine in a total volume of 0.25 ml. The concentrations of unlabelled drugs used are indicated in the figure legends. When progesterone or nicardipine were the competing drugs, dimethylsulphoxide (DMSO) was used as solvent to maintain solubility. Since [$^3$H]vinblastine binding is sensitive to DMSO, with an $EC_{50}$ of 2.5% (v/v), the assay volume for these drugs was increased to 1 ml, and 2 μl of the DMSO/drug solution used, reducing the final concentration of DMSO 0.2% (v/v). Vinblastine binding in the presence of each competing drug was corrected for the non-specific binding component (as defined above).

To measure the association kinetics of [$^3$H]vinblastine binding to P-gp, assays were carried out in the presence of 15–20 nM [$^3$H]vinblastine in a volume of 0.25 ml. Reactions were started by the addition of membranes (60–100 μg protein) followed by incubation for various times over a 180-min period prior to filtration. Binding to membranes was determined at each time point in the presence or absence of 3 μM vinblastine.

### Data analysis

Saturation binding curves for [$^3$H]vinblastine were analysed using the Langmuir Isotherm:

$$B_O = \{(B_{max} * F)/(K_d + F)\}$$

where $B_{max}$ = maximum density of binding sites (pmol mg$^{-1}$)
$K_d$ = dissociation constant (nM)
F = concentration of free [$^3$H]vinblastine (nM).

© 1999 Cancer Research Campaign

The ability of modulating drugs to displace [$^3$H]vinblastine binding from P-gp was analysed by non-linear least squares regression using the general dose–response equation (De Lean et al, 1978) defined as:

$$Y = \{(a–b)/(1+\{x/c\}^d)\} + b$$

where x = concentration of unlabelled drug (M)
a = maximum amount of [$^3$H]vinblastine bound
b = minimum amount of [$^3$H]vinblastine bound
d = slope factor
c = $IC_{50}$ concentration (M).

$IC_{50}$ values generated using this analysis were then used to calculate $K_i$ values from the Cheng and Prusoff equation (Cheng and Prusoff, 1973). The $K_i$ value is a measure of the inhibition constant for given drug whereas the $IC_{50}$ value varies with both the [$^3$H]vinblastine concentration and the amount of protein. The $K_i$ value is defined below.

$$K_i = IC_{50}/\ (1+[L]/K_d)$$

where $IC_{50}$ = concentration of drug required to inhibit 50% of the maximal binding of [$^3$H]vinblastine (M)
L = concentration of [$^3$H]vinblastine used (M)
$K_d$ = dissociation constant for [$^3$H]vinblastine binding (nM).

The association of [$^3$H]vinblastine with P-gp was modelled to the equation describing a second-order reversible reaction:

$$P + V \underset{k_{-1}}{\overset{k_{+1}}{\rightleftharpoons}} PV$$

where P = P-glycoprotein
V = [$^3$H]vinblastine
$k_{+1}$ = association rate constant (min$^{-1}$ nM$^{-1}$)
$k_{-1}$ = dissociation rate constant (min$^{-1}$).

The differential of this equation (see below) was fitted to the association data and the kinetic constants derived.

$$[PV] = \frac{[V]}{([V] + K_d)} \bullet \{1 - e^{-(k_{+1} \bullet [V] + k_{-1}) \bullet t}\}$$

All binding data were analysed by Kaleidagraph (Abelbeck Software). For all assays, means from *n* experiments are given with standard errors of the mean (s.e.m.). Statistical comparisons were made with the Student's *t*-test and a *P*-value of < 0.05 defined as statistically significant.

### RESULTS

### Expression of P-gp in LR73 cell lines

The LR73-1a and LR73-1b cell lines used in this study were derived from Chinese hamster ovary LR73 cells, stably transfected with the murine *mdr1a* or *mdr1b* genes respectively. In order to compare the levels of P-gp expressed in each cell line, plasma membranes were prepared, proteins (0.5–12 µg) separated by SDS-PAGE and P-gp detected by Western blotting (Figure 1). P-gp in membranes from LR73-1a and LR73-1b membranes ran as a diffuse band of 160–180 kDa. There was no detectable P-gp in the parental cell line, LR73 (data not shown). The slight difference in mobility between Pgp1a and Pgp1b is presumed due to the differential glycosylation between the two isoforms and has also been previously described (Valverde et al, 1996). The monoclonal antibody used, C219, has previously been demonstrated to detect rodent P-gp irrespective of the isoform (Georges et al, 1990).

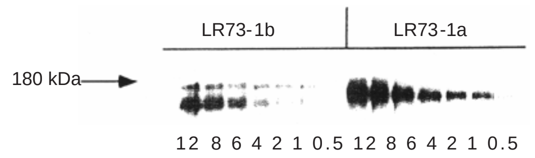

**Figure 1** Western immunoblot of membrane protein from the LR73-la and LR73-1b cell lines. Membrane protein (0.5–12 µg) was loaded onto 8% SDS-polyacrylamide gels and electrophoresed as described in Materials and Methods. The proteins were then transferred to nitrocellulose membranes and probed with the anti-Pgp monoclonal antibody C219

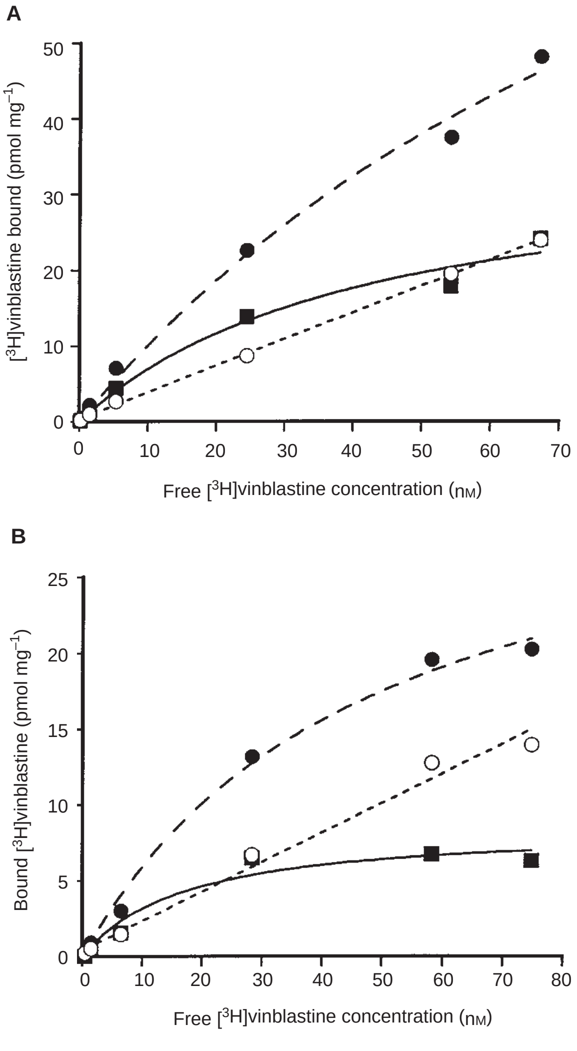

**Figure 2** Saturation isotherm of [$^3$H]vinblastine binding to membranes from the P-gp expressing cell lines LR73-1a (**A**) and LR73-1b (**B**). Membranes (60–100 µg protein) were incubated with [$^3$H]vinblastine (0–100 nM) for 120 min at 20°C, and bound and free ligand separated by rapid filtration. Total (●), and non-specific binding (○) was determined in the absence or presence of 3-µM unlabelled vinblastine respectively. The specific binding data (■) were obtained form a single representative experiment and parameters, obtained from curve fitting as described in Materials and Methods, were: $K_d$ = 41 ± 19 nM, $B_{max}$ = 36 ± 8 pmol mg$^{-1}$ for P-gp1a; and $K_d$ = 17 ± 9 nM, $B_{max}$ = 8.6 ± 1.4 pmol mg$^{-1}$ for P-gp1b

© 1999 Cancer Research Campaign

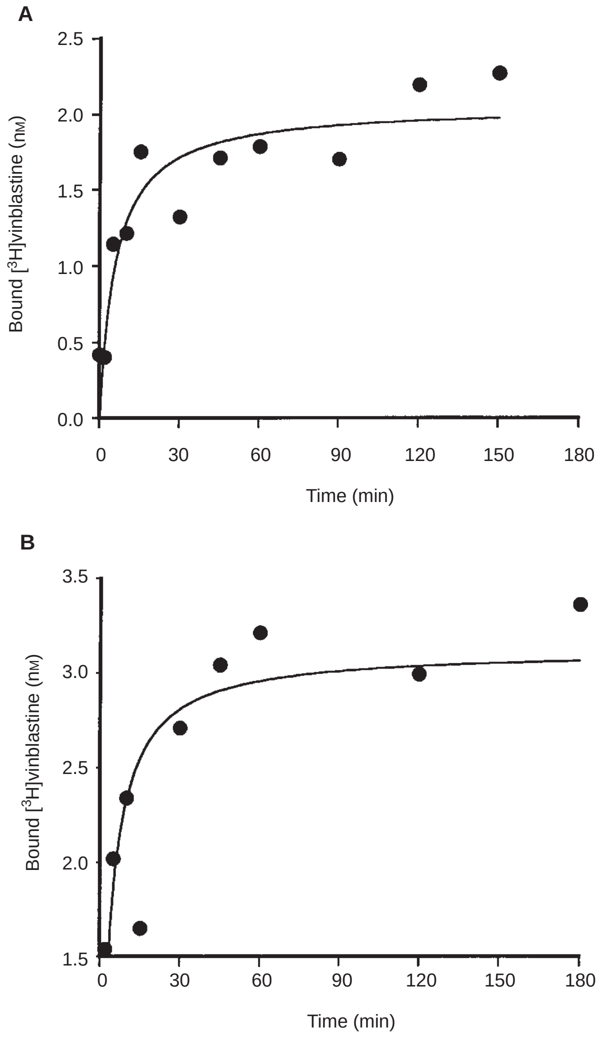

**Figure 3** Association kinetics of [$^3$H]vinblastine binding to membranes from the P-gp-expressing cell lines LR73-1a (**A**) and LR73-1b (**B**). Membranes (60–100 μg) were incubated with [$^3$H]vinblastine (15–20 nM) for the time indicated prior to filtration. Non-specific binding (in the presence of 3 μM unlabelled vinblastine) was subtracted from total binding and a single representative experiment is shown. The association rate constants were 0.002 ± 0.0005 and 0.002 ± 0.0006 min$^{-1}$ nM$^{-1}$ for LR73-1a and LR73-1b membranes respectively

Quantitation of the amount of P-gp in each band by densitometry revealed that the content of P-gp in LR73-1a membranes was approximately twice (1.8 ± 0.2-fold) that of the LR73-1b membranes.

### [$^3$H]Vinblastine binding to LR73-1a and LR73-1b membranes

[$^3$H]Vinblastine (0–100 nM) was shown to bind to membranes from both LR73-1a and LR73-1b cells in a specific fashion and the specific component of total binding displayed saturability (Figure 2). Non-specific binding of [$^3$H]vinblastine accounted for approximately 20% of the total binding. In contrast, [$^3$H]vinblastine did not bind specifically to the LR73 membranes that do not express P-gp (data not shown). These assays were carried out in hypotonic conditions in order to prevent the formation of membrane vesicles, and the accumulation of [$^3$H]vinblastine within such vesicles, which would give an overestimate of the binding capacity. The density of specific binding sites ($B_{max}$) on the LR73-1a membranes (24.5 ± 2.9 pmol mg$^{-1}$) was approximately twice that for the LR73-1b membranes (14.1 ± 2.9 pmol mg$^{-1}$). Since the data from the Western blots (see above) indicated that there was about 1.8 times the amount of P-gp in the LR73-1a membranes compared with the LR73-1b membranes, it appears that P-gp1a and P-gp1b have the same density of vinblastine-binding sites. The fraction of total membrane protein which P-gp represents can be estimated from the $B_{max}$ values, assuming that 1 mole of P-gp binds 1 mole of [$^3$H]vinblastine. The values obtained were 0.42% and 0.25% of total membrane protein for LR73-1a and LR73-1b membranes respectively. The affinity of [$^3$H]vinblastine binding was similar for LR73-1a ($K_d$ = 32.9 ± 4.9 nM) and LR73-1b (30.7 ± 7.6 nM) membranes, indicating similar drug interaction sites on the two isoforms. Direct comparison of the binding characteristics of [$^3$H]progesterone to P-gp was not possible due to (i) the poor affinity, low specific binding of this compound and (ii) the high non-specific component of total binding which was indicative of the hydrophobicity of this compound (data not shown).

### Association kinetics for [$^3$H]vinblastine binding to LR73-1a and LR73-1b membranes

The data above showed no difference in binding site properties between the P-gp1a and P-gp1b isoforms at equilibrium. Consequently, the rate at which [$^3$H]vinblastine associates with P-gp in LR73-1a and LR73-1b membranes was assessed. The amount of [$^3$H]vinblastine bound to the membranes as a function of time is shown in Figure 3. Analysis of these data generated rate constants ($k_{+1}$) of 0.002 ± 0.0005 and 0.002 ± 0.0006 min$^{-1}$ nM$^{-1}$ for LR73-1a and LR73-1b membranes respectively. Similarly the rate constants of [$^3$H]vinblastine dissociation (derived from Figure 3) from P-gp in LR73-1a (0.07 ± 0.02 min$^{-1}$) and LR73-1b (0.11 ± 0.02 min$^{-1}$) membranes were not significantly different. Therefore, no difference in the kinetics of [$^3$H]vinblastine binding to P-gp1a and P-gp1b, could be detected. The dissociation constant ($K_d$) for the formation of a drug–receptor complex is defined as $k_{-1}/k_{+1}$. Estimates of dissociation constants, using the kinetic data, were 35 nM and 55 nM for the P-gp1a and P-gp1b isoforms respectively, in reasonable agreement with the experimentally derived value at equilibrium.

### Displacement of [$^3$H]vinblastine binding by pharmacological agents

To further characterize the binding properties of the mouse P-gp isoforms, particularly the interaction of drugs at the vinblastine-binding site, several classes of displacing agent were used. The high affinity ligands, CP100-356 and nicardipine, the well-characterized MDR reversal agent verapamil, and the steroid hormone progesterone, were studied for their ability to displace specific [$^3$H]vinblastine binding from P-gp (Figure 4).

The order of potency for displacing vinblastine binding was CP100-356 > nicardipine > verapamil > progesterone. The diaminoquinazoline, CP100-356, is clearly a high affinity ligand for P-gp, with $K_i$ values of 58 ± 2 nM and 94 ± 21 nM for the P-gp1a and P-gp1b isoforms respectively. Nicardipine, a 1,4-dihydropyridine, which binds to a distinct site allosterically-linked to the vinblastine site (Malkhandi et al, 1994; Martin et al, 1997), displaced [$^3$H]-vinblastine binding to the P-gp1a isoform

© 1999 Cancer Research Campaign

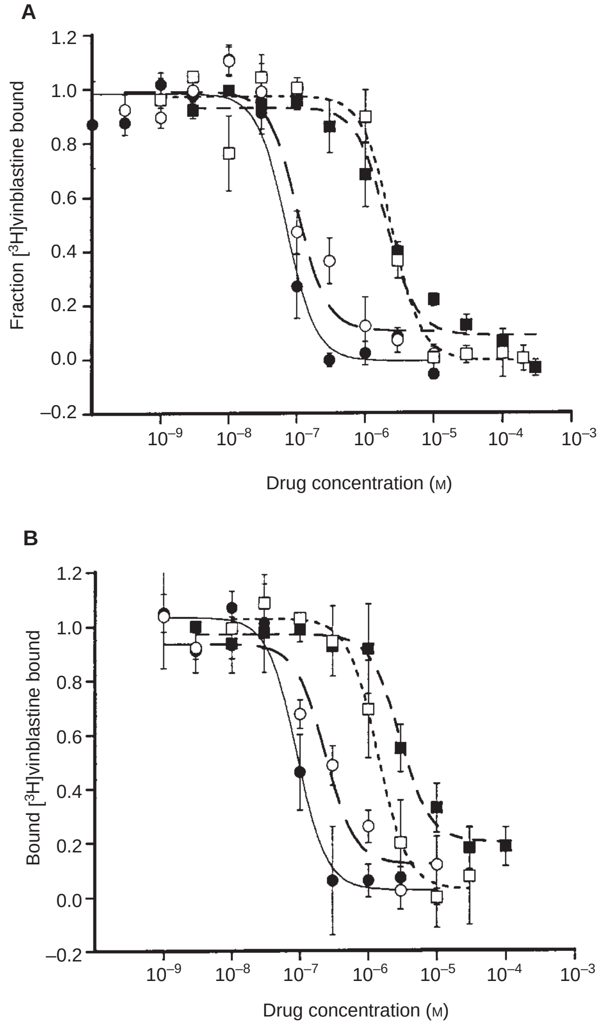

**Figure 4** The effects of CP100-356 (●), nicardipine (○), verapamil (■) and progesterone (□) on [$^3$H]vinblastine binding to membranes from the P-gp-expressing cell lines LR73-1a (**A**) and LR73-1b (**B**). Drugs were added at the concentrations indicated in the presence of [$^3$H]vinblastine (15–20 nM), and equilibrated for 120 min prior to filtration. Non-specific binding was determined in the presence of 3-μM unlabelled vinblastine. The values (mean ± s.e.m.) were obtained from 3–5 independent membrane preparations

($K_i$ = 128 ± 20 nM) with approximately twice the affinity observed for P-gp1b-containing membranes ($K_i$ = 239 ± 48 nM; $P < 0.05$). In contrast to CP100-356, progesterone was found to have a low and almost identical affinity for both isoforms ($K_i$ = 1.9 ± 0.3 μM and 1.8 ± 0.2 μM for P-gp1a and P-gp1b respectively). Verapamil displayed a threefold higher affinity for P-gp1a compared with P-gp1b ($K_i$ = 1200 ± 200 nM and 3000 ± 500 nM; $n = 4$), a modest difference, but one which is statistically significant at the 2% level ($P < 0.02$).

In summary, the vinblastine binding site(s) on P-gp1a and P-gp1b are equivalent in terms of affinity for substrate and the kinetics of its association/dissociation. The only pharmacological differences observed were small differences in the ability of CP100-356 and verapamil to displace vinblastine from its binding site, perhaps indicating small differences in its structure.

### DISCUSSION

Despite their strong sequence similarity, the complementary patterns of tissue-specific expression suggest that the mouse isoforms of P-gp may be functionally distinct (Arceci et al, 1988; Croop et al, 1989; Piekarz et al, 1993; Schinkel et al, 1994). In particular, the expression patterns of the P-gp1b isoform suggest it may play a specific role in steroid transport (Altuvia et al, 1993). Consistent with these ideas, several studies suggest that cells expressing the P-gp1a and P-gp1b isoforms differ in their resistance to certain drugs (Yang et al, 1990; Kajiji et al, 1993; Tang-Wai et al, 1995). However, such cytotoxicity assays provide an indirect assay for drug transport, and differences in cytotoxicity may be caused by factors other than the transport properties of P-gp itself. Thus, in this study we directly assayed the interaction of a variety of compounds, including progesterone, with the murine P-gp1a and P-gp1b isoforms.

No difference could be detected in the affinity or binding capacity, at equilibrium, of the two isoforms towards the vinca alkaloid vinblastine. Similarly, using kinetic techniques, the rate at which vinblastine associates with the drug binding site was identical for both P-gp1a and P-gp1b. However, a previous manuscript has suggested that P-gp1a is a more 'efficient' [$^3$H]vinblastine transporter than Pgp1b (Yang et al, 1990). This difference between binding and transport data suggests that the isoforms differ in substrate handling at a point subsequent to the initial binding interaction. It is possible that coupling between binding and ATP hydrolysis, which is required for transport, or perhaps a step in the translocation pathway differs between the P-gp1a and P-gp1b isoforms. Both studies indicate similar binding affinity and capacity of the isoforms towards P-gp; however, the data obtained from Yang et al (1990) suggested that the P-gp1a isoform contains a second [$^3$H]vinblastine-binding site. Our data show no evidence for more than one vinblastine-binding site on either P-gp isoform. The presence of two binding sites was derived following linear transformation of the binding data using the Scatchard technique. This type of analysis has been questioned and proved statistically invalid (Burgisser, 1984; Kenakin, 1997). Using the now preferred technique of non-linear regression we were unable to detect the presence of multiple binding sites on P-gp for vinblastine.

A further approach to characterizing drug binding by the two isoforms of P-gp was the use of agents to displace vinblastine binding. Several lines of evidence measuring transport, binding and ATPase activity of P-gp have pointed to the presence of multiple drug interaction sites (Ferry et al, 1992, 1995; Spoelstra et al, 1992; Malkhandi et al, 1994; Ayesh et al, 1996; Orlowski et al, 1996; Martin et al, 1997). CP100-356 is a diaminoquinazoline and amongst the most potent MDR-modulating agents reported (Kajiji et al, 1994), hence providing specificity and selectivity of binding displacement. The modest difference in the affinity of P-gp1a and P-gp1b for CP100-356, estimated by its ability to displace vinblastine binding, and similar modest differences for verapamil, are unlikely to account for the duplication of the two drug transporting P-gp isoforms in mouse. Nicardipine, a 1,4-dihydropyridine, in contrast to CP100-356 and verapamil, increases the dissociation rate for vinblastine implying that it interacts with a distinct but allosterically-linked binding site. The similar potency with which nicardipine displaced vinblastine binding from P-gp1a and P-gp1b shows that the two isoforms are unlikely to differ significantly in this second binding site or in communication between the two allosterically-linked sites.

*© 1999 Cancer Research Campaign* 

Based on tissue-specific patterns of expression, and a progesterone response element in the *mdr1b* promoter region, a physiological role in steroid transport has been suggested for P-gp1b. However, we were unable to demonstrate any difference in the ability of progesterone to affect vinblastine binding to P-gp1a and P-gp1b. Previous reports, using a range of less direct assay techniques, also failed consistently to distinguish between the two mouse isoforms in terms of steroid binding or transport properties (Ueda et al, 1992; Barnes et al, 1996). Thus, the present data suggest that differences in the ability to transport progesterone are unlikely to account for the duplication giving rise to P-gp1a and P-gp1b. In addition, the $K_i$ value for progesterone binding to P-gp determined here demonstrates a sufficiently poor affinity that progesterone is unlikely to be a physiological substrate in vivo. It seems more likely that progesterone regulates the relative cellular expression levels of the two isoforms of P-gp.

In conclusion, we have found no significant differences between the P-gp1a and P-gp1b isoforms in the nature of drug binding sites or communication between allosterically linked regulatory sites. Therefore, if biochemical differences exist between the isoforms they must be at a later step in transport such as the translocation step or linkage between drug binding and ATP hydrolysis. The possibility that the gene duplication is of no functional significance seems unlikely given its conservation in all rodents examined. Alternatively, the duplication may reflect a function for P-gp distinct from its drug transport properties. In this regard, P-gp has been shown to modulate the activation of cell swelling-activated chloride currents, an activity distinct and separable from its function as a drug transporter (Gill et al, 1992; Hardy et al, 1995; Valverde et al, 1996). Interestingly, the two murine isoforms of P-gp differ in their ability to modulate channel activity; the P-gp1a isoform, like human P-gp increases the rate of activation of chloride currents while, in contrast, P-gp1b appears to have no effect (Valverde et al, 1996).

The P-gp1a and P-gp1b isoforms appear to differ with respect to: (i) modulating cell volume activated chloride currents (Valverde et al, 1996), (ii) cellular efflux of [$^3$H]vinblastine (Yang et al, 1990) and (iii) tissue distribution profiles (Croop et al, 1989). However, in the present manuscript we have detailed that the initial drug binding event/interaction between the two isoforms and several substrates is identical. Combined these pieces of evidence suggest that the presence of multiple isoforms of P-gp allows subtle quantitative and qualitative regulation of its respective cellular activity.

### ACKNOWLEDGEMENTS

We are grateful to the ICRF Clare Hall Laboratories for cell growth and to Phillipe Gros (Montreal) for LR73, LR73-1a and LR73-1b cells. This work was supported by the Cancer Research Campaign and the Imperial Cancer Research Fund. CFH is a Howard Hughes International Research Scholar.

### REFERENCES

Altuvia S, Stein WD, Goldenberg S, Kane SE, Pastan I and Gottesman MM (1993) Targeted disruption of the mouse *mdr1b* gene reveals that steroid hormones enhance *mdr* gene expression. *J Biol Chem* **268**: 27127–27132

Arceci RJ, Croop JM, Horwitz SB and Housman D (1988) The gene encoding multidrug resistance is induced and expressed at high levels during pregnancy in the secretory epithelium of the uterus. *Proc Natl Acad Sci USA* **85**: 4350–4354

Ayesh S, Shao Y-M and Stein WD (1996) Co-operative, competitive and non-competitive interactions between modulators of P-glycoprotein. *Biochim Biophys Acta* **1316**: 8–18

Barnes KM, Dickstein B, Cutler GB, Fojo T and Bates SE (1996) Steroid transport, accumulation and antagonism of P-glycoprotein in multidrug resistant cells. *Biochemistry* **35**: 4820–4827

Burgisser E (1984) Radioligand-receptor binding studies; what's wrong with the Scatchard analysis? *Trends Biochem Sci* 142–144

Cheng Y and Prusoff WH (1973) Relationship between the inhibition constant ($K_i$) and the concentration of inhibitor which causes 50% inhibition ($IC_{50}$) of an enzymic reaction. *Biochem Pharmacol* **22**: 3099–3108

Croop JM, Raymond M, Haber D, Devault A, Arceci RJ, Gros P and Housman DE (1989) The three mouse multidrug resistance (mdr) genes are expressed in a tissue-specific manner in normal mouse tissues. *Mol Cell Biol* **9**: 1346–1350

De Lean A, Munson PJ and Rodbard D (1978) Simultaneous analysis of families of sigmoidal curves: applications to bioassay, radioligand assay and physiological dose response curves. *J Physiol* **235**: E97–E102

Devault A and Gros P (1990) Two members of the mouse *mdr* gene family confer multidrug resistance with overlapping but distinct drug specificities. *Mol Cell Biol* **10**: 1652–1663

Ferry DR, Russell MA and Cullen MH (1992) P-glycoprotein possesses a 1,4-dihydropyridine selective drug acceptor site which is allosterically coupled to a vinca alkaloid selective binding site. *Biochem Biophys Res Commun* **188**: 440–445

Ferry DR, Malkhandi JP, Russell MA and Kerr DJ (1995) Allosteric regulation of [3H]vinblastine binding to P-glycoprotein of MCF-7 Adr cells by dexniguldipine. *Biochem Pharmacol* **49**: 1851–1861

Garrigos M, Mir LM and Orlowski S (1997) Competitive and non-competitive inhibition of the multidrug resistance-associated p-glycoprotein atpase: further experimental evidence for a multisite model. *Eur J Biochem* **244**: 644–673

Georges E, Bradley G, Gariepy J and Ling V (1990) Detection of P-glycoprotein isoforms by gene specific monoclonal antibodies. *Proc Natl Acad Sci USA* **87**: 152–156

Gill DR, Hyde SC, Higgins CF, Valverde MA, Mintenig, GM and Sepulveda FV (1992) Separation of drug transport and chloride channel functions of the human multidrug resistance P-glycoprotein. *Cell* **71**: 23–32

Gottesman MM and Pastan I (1988) The multi-drug transporter: a double edged sword. *J Biol Chem* **263**: 12163–12166

Gros P, Croop J and Housman D (1986) Mammalian multidrug resistance gene: complete cDNA sequence indicates strong homology to bacterial transport proteins. *Cell* **47**: 371–380

Hardy SP, Goodfellow HR, Valverde MA, Gill DR, Sepulveda FV and Higgins CF (1995) Protein kinase C-mediated phosphorylation of the human multidrug resistance P-glycoprotein regulates cell volume-activated chloride channels. *EMBO J* **14**: 68–75

Kajiji S, Talbot F, Grizzuti K, Van Dyke-Phillips V, Agresti M, Safa A and Gros P (1993) Functional analysis of P-glycoprotein mutants identifies predicted transmembrane domain 11 as a putative drug binding site. *Biochemistry* **32**: 4185–4194

Kajiji S, Dreslin JA, Grizzuti K and Gros P (1994) Structurally distinct MDR modulators show specific patterns of reversal against P-glycoproteins bearing unique mutations at serine$^{939/941}$. *Biochemistry* **33**: 5041–5048

Kenakin T (1997) In Pharmacologic Analysis of Drug-Receptor Interaction pp. 198–241. Lippincott-Raven, Philadelphia

Lever JE (1977) Active amino-acid transport in plasma membrane vesicles from Simian virus 40-transformed mouse fibroblasts. Characteristics of electrochemical Na+ gradient stimulated uptake. *J Biol Chem* **252**: 1990–1997

Malkhandi J, Ferry DR, Boer R, Gekeler V, Ise W and Kerr DJ (1994) Dexniguldipine-HCl is a potent allosteric inhibitor [$^3$H]vinblastine binding to P-glycoprotein of CCRF ADR5000 cells. *Eur J Pharmacol* **288**: 105–114

Martin C, Berridge G, Higgins CF and Callaghan R (1997) The multidrug resistance reversal agent SR33557 and modulation of vinca alkaloid binding to P-glycoprotein by an allosteric interaction. *British J Pharmacol* **121**: in press

Orlowski S, Mir LM, Belehradek JR and Garrigos M (1996) Effects of steroids and verapamil on P-glycoprotein ATPase activity: progesterone, desoxycorticosterone, corticosterone and verapamil are mutually non-exclusive modulators. *Biochem J* **317**: 515–522

Piekarz RL, Cohen D and Horwitz SB (1993) Progesterone regulates the murine multidrug resistance mdr1b gene. *J Biol Chem* **268**: 7613–7616

Schinkel AH, Smit JJ, van Tellingen O, Beijnen JH, Wagenaar E, van Deemter L, Mol CAAM, van der Valk MA, Robanus-Maandag EC, te Riele HPJ, Berns JM and Borst P (1994) Disruption of the mouse *mdr1a* P-glycoprotein gene leads

 *© 1999 Cancer Research Campaign*

to a deficiency in the blood–brain barrier and to increased sensitivity to drugs. *Cell* **77**: 491–502

Spoelstra EC, Westerhoff HV, Dekker H and Lankelma J (1992) Kinetics of doxorubicin transport by P-glycoprotein of intact cancer cells. *Eur J Biochem* **207**: 567–579

Tang-Wai DF, Kajiji S, DiCapua F, de Graaf D, Roninson IB and Gros P (1995) Human (MDR1) and mouse (*mdr1, mdr3*) P-glycoproteins can be distinguished by their respective drug resistance profiles and sensitivity to modulators. *Biochemistry* **34**: 32–39

Towbin H, Staehlin T and Gordon J (1979) Electrophoretic transfer of proteins from polyacrylamide gels to nitrocellulose sheets: procedure and some applications. *Proc Natl Acad Sci USA* **76**: 4350–4354

Trezise AEO, Romano PR, Gill DR, Hyde SC, Sepulveda FV, Buchwald M and Higgins CF (1992) The multidrug resistance and cystic-fibrosis genes have complementary patterns of epithelial expression. *EMBO J* **11**: 4291–4303

Ueda K, Okamura N, Hirai M, Tanigawara Y, Saeki T, Kioka N, Komano T and Hori R (1992) Human P-glycoprotein transports cortisol, aldosterone, but not progesterone. *J Biol Chem* **267**: 24248–24252

Valverde MA, Bond TD, Hardy SP, Taylor JC, Higgins CF, Altamirano J and Alvarez-Leefmans FJ (1996) The multidrug resistance P-glycoprotein modulates cell regulatory volume decrease. *EMBO J* **15**: 4460–4468

Yang CP, Cohen D, Greenberger LM, Hsu SI and Horwitz SB (1990) Differential transport properties of two mdr gene products are distinguished by progesterone. *J Biol Chem* **265**: 10282–10288

*© 1999 Cancer Research Campaign*

## References

[PDF_00675] Altuvia S., Stein W. D., Goldenberg S., Kane S. E., Pastan I., Gottesman M. M. (1993). Targeted disruption of the mouse mdr1b gene reveals that steroid hormones enhance mdr gene expression.. J Biol Chem.

[PDF_00678] Arceci R. J., Croop J. M., Horwitz S. B., Housman D. (1988). The gene encoding multidrug resistance is induced and expressed at high levels during pregnancy in the secretory epithelium of the uterus.. Proc Natl Acad Sci U S A.

[PDF_00682] Ayesh S., Shao Y. M., Stein W. D. (1996). Co-operative, competitive and non-competitive interactions between modulators of P-glycoprotein.. Biochim Biophys Acta.

[PDF_00685] Barnes K. M., Dickstein B., Cutler G. B., Fojo T., Bates S. E. (1996). Steroid treatment, accumulation, and antagonism of P-glycoprotein in multidrug-resistant cells.. Biochemistry.

[PDF_00695] Croop J. M., Raymond M., Haber D., Devault A., Arceci R. J., Gros P., Housman D. E. (1989). The three mouse multidrug resistance (mdr) genes are expressed in a tissue-specific manner in normal mouse tissues.. Mol Cell Biol.

[PDF_00698] DeLean A., Munson P. J., Rodbard D. (1978). Simultaneous analysis of families of sigmoidal curves: application to bioassay, radioligand assay, and physiological dose-response curves.. Am J Physiol.

[PDF_00701] Devault A., Gros P. (1990). Two members of the mouse mdr gene family confer multidrug resistance with overlapping but distinct drug specificities.. Mol Cell Biol.

[PDF_00708] Ferry D. R., Malkhandi P. J., Russell M. A., Kerr D. J. (1995). Allosteric regulation of [3H]vinblastine binding to P-glycoprotein of MCF-7 ADR cells by dexniguldipine.. Biochem Pharmacol.

[PDF_00704] Ferry D. R., Russell M. A., Cullen M. H. (1992). P-glycoprotein possesses a 1,4-dihydropyridine-selective drug acceptor site which is alloserically coupled to a vinca-alkaloid-selective binding site.. Biochem Biophys Res Commun.

[PDF_00711] Garrigos M., Mir L. M., Orlowski S. (1997). Competitive and non-competitive inhibition of the multidrug-resistance-associated P-glycoprotein ATPase--further experimental evidence for a multisite model.. Eur J Biochem.

[PDF_00715] Georges E., Bradley G., Gariepy J., Ling V. (1990). Detection of P-glycoprotein isoforms by gene-specific monoclonal antibodies.. Proc Natl Acad Sci U S A.

[PDF_00718] Gill D. R., Hyde S. C., Higgins C. F., Valverde M. A., Mintenig G. M., Sepúlveda F. V. (1992). Separation of drug transport and chloride channel functions of the human multidrug resistance P-glycoprotein.. Cell.

[PDF_00721] Gottesman M. M., Pastan I. (1988). The multidrug transporter, a double-edged sword.. J Biol Chem.

[PDF_00723] Gros P., Croop J., Housman D. (1986). Mammalian multidrug resistance gene: complete cDNA sequence indicates strong homology to bacterial transport proteins.. Cell.

[PDF_00726] Hardy S. P., Goodfellow H. R., Valverde M. A., Gill D. R., Sepúlveda V., Higgins C. F. (1995). Protein kinase C-mediated phosphorylation of the human multidrug resistance P-glycoprotein regulates cell volume-activated chloride channels.. EMBO J.

[PDF_00734] Kajiji S., Dreslin J. A., Grizzuti K., Gros P. (1994). Structurally distinct MDR modulators show specific patterns of reversal against P-glycoproteins bearing unique mutations at serine939/941.. Biochemistry.

[PDF_00730] Kajiji S., Talbot F., Grizzuti K., Van Dyke-Phillips V., Agresti M., Safa A. R., Gros P. (1993). Functional analysis of P-glycoprotein mutants identifies predicted transmembrane domain 11 as a putative drug binding site.. Biochemistry.

[PDF_00740] Lever J. E. (1977). Active amino acid transport in plasma membrane vesicles from Simian virus 40-transformed mouse fibroblasts. Characteristics of electrochemical Na+ gradient-stimulated uptake.. J Biol Chem.

[PDF_00744] Malkhandi J., Ferry D. R., Boer R., Gekeler V., Ise W., Kerr D. J. (1994). Dexniguldipine-HCl is a potent allosteric inhibitor of [3H]vinblastine binding to P-glycoprotein of CCRF ADR 5000 cells.. Eur J Pharmacol.

[PDF_00751] Orlowski S., Mir L. M., Belehradek J., Garrigos M. (1996). Effects of steroids and verapamil on P-glycoprotein ATPase activity: progesterone, desoxycorticosterone, corticosterone and verapamil are mutually non-exclusive modulators.. Biochem J.

[PDF_00755] Piekarz R. L., Cohen D., Horwitz S. B. (1993). Progesterone regulates the murine multidrug resistance mdr1b gene.. J Biol Chem.

[PDF_00764] Spoelstra E. C., Westerhoff H. V., Dekker H., Lankelma J. (1992). Kinetics of daunorubicin transport by P-glycoprotein of intact cancer cells.. Eur J Biochem.

[PDF_00767] Tang-Wai D. F., Kajiji S., DiCapua F., de Graaf D., Roninson I. B., Gros P. (1995). Human (MDR1) and mouse (mdr1, mdr3) P-glycoproteins can be distinguished by their respective drug resistance profiles and sensitivity to modulators.. Biochemistry.

[PDF_00771] Towbin H., Staehelin T., Gordon J. (1979). Electrophoretic transfer of proteins from polyacrylamide gels to nitrocellulose sheets: procedure and some applications.. Proc Natl Acad Sci U S A.

[PDF_00774] Trezise A. E., Romano P. R., Gill D. R., Hyde S. C., Sepúlveda F. V., Buchwald M., Higgins C. F. (1992). The multidrug resistance and cystic fibrosis genes have complementary patterns of epithelial expression.. EMBO J.

[PDF_00777] Ueda K., Okamura N., Hirai M., Tanigawara Y., Saeki T., Kioka N., Komano T., Hori R. (1992). Human P-glycoprotein transports cortisol, aldosterone, and dexamethasone, but not progesterone.. J Biol Chem.

[PDF_00780] Valverde M. A., Bond T. D., Hardy S. P., Taylor J. C., Higgins C. F., Altamirano J., Alvarez-Leefmans F. J. (1996). The multidrug resistance P-glycoprotein modulates cell regulatory volume decrease.. EMBO J.

[PDF_00783] Yang C. P., Cohen D., Greenberger L. M., Hsu S. I., Horwitz S. B. (1990). Differential transport properties of two mdr gene products are distinguished by progesterone.. J Biol Chem.

